# Sleep Quality Mediates the Effect of Sensitization-Associated Symptoms, Anxiety, and Depression on Quality of Life in Individuals with Post-COVID-19 Pain

**DOI:** 10.3390/brainsci12101363

**Published:** 2022-10-08

**Authors:** Juan C. Pacho-Hernández, César Fernández-de-las-Peñas, Stella Fuensalida-Novo, Carmen Jiménez-Antona, Ricardo Ortega-Santiago, Margarita Cigarán-Mendez

**Affiliations:** 1 Department of Psychology, Universidad Rey Juan Carlos, 28922 Alcorcón, Spain; 2 Department of Physical Therapy, Occupational Therapy, Physical Medicine and Rehabilitation, Universidad Rey Juan Carlos (URJC), 28922 Alcorcón, Spain

**Keywords:** post-COVID-19, sensitization, depression, anxiety, sleep, quality of life

## Abstract

A better understanding of biological and emotional variables associated with health-related quality of life in people with long-COVID is needed. Our aim was to identify potential direct and indirect effects on the relationships between sensitization-associated symptoms, mood disorders such as anxiety/depressive levels, and sleep quality on health-related quality of life in people suffering from post-COVID-19 pain. One hundred and forty-six individuals who were hospitalized due to COVID-19 during the first wave of the pandemic and suffering from long-term post-COVID-19 pain completed different patient-reported outcome measures (PROMs), including clinical features, symptoms associated with sensitization of the central nervous system (Central Sensitization Inventory), mood disorders (Hospital Anxiety and Depressive Scale), sleep quality (Pittsburgh Sleep Quality Index), and health-related quality of life (paper-based five-level version of EuroQol-5D) in a face-to-face interview conducted at 18.8 (SD 1.8) months after hospitalization. Different mediation models were conducted to assess the direct and indirect effects of the associations among the different variables. The mediation models revealed that sensitization-associated symptoms and depressive levels directly affected health-related quality of life; however, these effects were not statistically significant when sleep quality was included. In fact, the effect of sensitization-associated symptomatology on quality of life (β = −0.10, 95% CI −0.1736, −0.0373), the effect of depressive levels on quality of life (β= −0.09, 95% CI −0.1789, −0.0314), and the effect of anxiety levels on quality of life (β = −0.09, 95% CI −0.1648, −0.0337) were all indirectly mediated by sleep quality. This study revealed that sleep quality mediates the relationship between sensitization-associated symptoms and mood disorders (depressive/anxiety levels) with health-related quality of life in individuals who were hospitalized with COVID-19 at the first wave of the pandemic and reporting post-COVID-19 pain. Longitudinal studies will help to determine the clinical implications of these findings.

## 1. Introduction

The severe acute respiratory syndrome coronavirus 2 (SARS-CoV-2) virus, responsible for coronavirus disease 2019 (COVID-19), is primarily considered a virus affecting the respiratory system; however, a multisystemic affection including neurological, gastrointestinal, and cardiological manifestations, as well as multiple organ failure, have been seen in severe COVID-19 patients [1]. The affectation of different systems leads to a plethora of symptoms during the acute phase of the infection such as fever, dyspnea, throat pain, diarrhea, chest pain, confusion, and others [2]. The appearance of the SARS-CoV-2 virus has represented a worldwide sanitary crisis due to the billions of infected individuals and the millions of deaths.

Additionally, COVID-19 has evolved to a second outbreak, i.e., the presence of long-lasting symptoms several weeks or months after surviving SARS-CoV-2 acute infection, a condition called long-COVID [3] or post-COVID-19 [4]. Evidence supports the idea that almost 60% of COVID-19 survivors experience different post-COVID symptoms, at least during the first year after the acute infection [5,6,7]. More than 50 post-COVID-19 symptoms have been described, with fatigue, dyspnea, pain, or cognitive problems being the most prevalent [8].

Patients with post-COVID-19 report lower health-related quality of life (HRQoL) [9] and a reduction of daily living activities [10]. Soriano et al., in a Delphi consensus study, have recently included functional repercussions into the definition of the post-COVID-19 condition: “…these symptoms generally have an impact on everyday function …” [4]. In fact, increasing evidence shows the potential burden for healthcare systems that post-COVID-19 represents [11]. Accordingly, identification of factors associated with HRQoL in people with the post-COVID-19 condition is essential.

Pain is an underestimated post-COVID-19 symptom that can generate a potential burden to the society [12]. The prevalence rate of post-COVID-19 pain symptomatology ranges between 15% and 50%, depending on the study [13,14]. Post-COVID-19 pain symptoms can exhibit musculoskeletal [15] or neuropathic [16] features in 45% and 25% of the subjects, respectively. Musculoskeletal post-COVID-19 pain is perceived in any part of the body, but the upper and lower extremities are the most prevalent locations [14,15]. Others have reported incidence rates ranging from 1.7% to 33.9% for headache, from 1.5% to 61% for myalgia or arthralgia, from 1.6% to 17.7% for chest pain, and from 1.9% to 14.5% for abdominal pain [17]. The most accepted theory explaining post-COVID-19 pain is that the systemic inflammatory–immune response (i.e., long-lasting cytokine storm) can lead to an excitability of peripheral/central nervous systems throughout direct/indirect pain pathways [18,19]. A prolonged excitability of the nervous system could promote the presence of sensitization mechanisms in these patients. In fact, it has been hypothesized that post-COVID-19 pain could be classified as a “nociplastic condition”. Nociplastic pain is defined as “pain that arises from altered nociception without clear evidence of actual or threatened tissue damage causing the activation of peripheral nociceptors or evidence for disease or lesion of the somatosensory system causing pain” [20]. The first rational for considering post-COVID-19 pain as a nociplastic condition is the increasing evidence suggesting the presence of sensitization, the basis of nociplastic pain, in individuals with post-COVID-19 pain [21,22]. Another rational for considering post-COVID-19 pain as a nociplastic condition is the presence of central-nervous-system-derived symptoms, e.g., fatigue, sleep problems, depression, anxiety, and memory loss, which are common in nociplastic pain [23] and also in individuals with the post-COVID-19 condition [5,6,7]. Finally, some studies have also revealed that almost 60% of individuals with post-COVID-19 pain exhibit multiple pain sites [24] and that also 30% of patients share clinical features with fibromyalgia syndrome [25].

In addition to physical symptoms, people with the post-COVID-19 condition also present emotional symptomatology. Anxiety and depressive symptoms are prevalent at the acute phase of SARS-CoV-2 infection [26,27] but also at the post-COVID-19 phase [28]. The presence of physical and emotional symptoms in patients with the post-COVID-19 condition supports the observation that biological and behavioral factors interact in a COVID-19 context [29]. It has been recently found that higher depressive symptomatology is associated with a higher risk of physical post-COVID-19 symptoms such as pain and dyspnea [30]. In fact, the interaction between anxiety and depressive levels with pain is supported in the literature since mood disorders can contribute to chronic pain via supra-spinal mechanisms and emotional modulation of pain [31]. Therefore, a better understanding of the interactions between sensitization-associated symptoms, mood disorders, sleep, and HRQoL in people with post-COVID-19 pain may assist clinicians to determine better therapeutic programs for this population.

Accordingly, the purpose of this study was to identify the direct and indirect effects on the relationships between sensitization-associated symptoms and emotional disorders on HRQoL in a sample of previously hospitalized COVID-19 survivors with long-term post-COVID-19 pain.

## 2. Methods

### 2.1. Participants

From all individuals hospitalized during the first wave of the COVID-19 pandemic (March–April 2020) due to an acute SARS-CoV-2 infection from three urban hospitals in Madrid (Spain), a randomly sample of 200 individuals were included into an anonymous electronic database and invited to participate in a face-to-face interview. To be selected, patients should fulfill the following criteria: (1) diagnosis of SARS-CoV-2 infection by real-time reverse transcription-polymerase chain reaction (RT-PCR) assay of nasopharyngeal/oral swab sample and clinical and radiological findings at hospital admission; (2) hospitalization during the first wave of the pandemic; (3) presence of post-COVID-19 pain symptoms starting no later than two months after hospitalization and lasting for at least three months [4]; and (4) absence of underlying medical conditions that could best explain pain, e.g., arthritis. Exclusion criteria consisted of (1) pre-existing history of chronic pain symptoms before SARS-CoV-2 infection and (2) pre-existing medical comorbidity explaining pain symptoms.

This study was approved by all the Ethics Committees of all the involved institutions (INDIVAL Cantabria 2020.416, HUFA 20/126, URJC0907202015920, HSO25112020, HUIL/092-20). All participants provided their written informed consent prior to their inclusion.

Participants were scheduled for a face-to-face interview with an experienced assessor where they fulfilled demographic data (i.e., age, sex, height, and weight), clinical data (i.e., intensity and duration of pain), and the following PROMS: sensitization-associated symptoms, anxiety and depressive symptoms (considered as the independent variables), sleep quality (considered as the mediating variable), and health-related quality of life (considered as the dependent variable). Data from hospitalization were obtained from medical records.

### 2.2. Dependent Variable: Health-Related Quality of Life

Health-related quality of life (HRQoL) was assessed with the paper-based five-level version of EuroQol-5D (EQ-5D-5L) [32]. This PROM assesses the following items: mobility, self-care, daily life activities, pain symptoms, and depression/anxiety into 5-point Likert scales ranging from 0 (no problem) to 4 (severe problems) points. Responses are converted into a single index number ranging from 0 (health state judged to be equivalent to death) to 1 (optimal health) by applying crosswalk index values for Spain life [33]. The EQ-5D-5L has shown excellent test–retest reliability in individuals with post-COVID-19 (ICC 0.86) [32].

### 2.3. Mediating Variable: Sleep Quality

The Pittsburgh Sleep Quality Index (PSQI) was the PROM used to assess sleep quality [34]. This PROM includes 19 questions assessing different aspects of sleep, e.g., usual bedtime, wake-up time, number of hours slept, and time needed to fall asleep in a 4-point Likert scale ranging from 0 to 3 points. The total score ranges from 0 to 21 points, where ≥8.0 points are indicative of being poor sleeper [34]. The PSQI has exhibited excellent test–retest reliability patients with primary insomnia (ICC 0.87) [35].

### 2.4. Independent Variables: Sensitization-Associated Symptoms and Mood Disorders

The Central Sensitization Inventory (CSI) was used to evaluate the presence of sensitization-associated symptomatology [36]. This PROM evaluates the presence of 25 symptoms associated with sensitization on a 5-point Likert scale. The total score ranges from 0 to 100 points. A score > 40 points suggests the presence of symptoms related to sensitization [37]. The CSI has also shown excellent reliability (ICC 0.95) and good psychometric properties in individuals with persistent pain [38,39].

Anxiety and depressive symptoms were assessed with the Hospital Anxiety and Depression Scale (HADS) [40]. Each scale includes 7 items evaluating anxiety (HADS-A) or depressive (HADS-D) symptoms on a 4-point Likert scale (0–3). The score of each subscale ranges from 0 to 21 points, where ≥12 points on the HADS-A are indicative of anxiety symptoms and ≥10 points on the HADS-D indicate of depressive symptoms [41]. A recent study has revealed that both scales of the HADS exhibit good internal consistency in people with the post-COVID-19 condition [40].

### 2.5. Statistical Analysis

**Figure 1:**
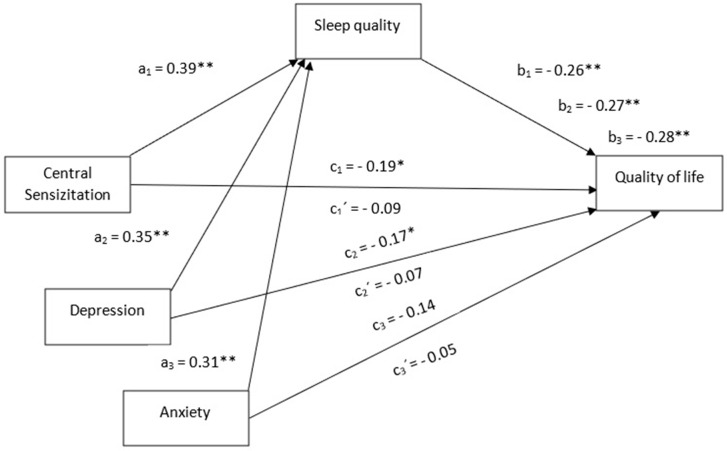
Mediation models with standardized coefficients. Model 1. a_1_ = beta value of sensitization-associated symptoms on sleep quality; b_1_ = beta value of sleep quality on quality of life; c_1_ = total effect of sensitization-associated symptoms on quality of life; c_1_’ = direct effect of sensitization-associated symptoms on quality of life once sleep quality is included in the model. Model 2. a_2_ = beta value of depressive levels on sleep quality; b_2_ = beta value of sleep quality on quality of life; c_2_ = total effect of depressive levels on quality of life; c_2_’ = direct effect of depressive levels on quality of life once sleep quality has been included in the model. Model 3. a_3_ = beta value of anxiety on sleep quality; b_3_ = beta value of sleep quality on quality of life; c_3_= total effect of anxiety on quality of life; c_3_’ = direct effect of anxiety on quality of life once sleep quality has been included in the model. * *p* < 0.05; ** *p* < 0.01.

Descriptive (means, standard deviations) and frequency analyses were calculated using the statistical program IBM SPSS 27.0. In addition, the PROCESS macro version 4.1 [42] for SPSS was used for testing the simple mediation models depicted in Figure 1, as well as for calculating total and direct effects of each independent variable (sensitization-associated symptoms, depressive levels, anxiety levels) on the dependent variable (HRQoL), and also the indirect effect of sleep quality (the proposed mediating variable) in the relationship between each independent variable with the dependent variable.

PROCESS uses a bootstrapping method that allows for the calculation of the total effect, the direct effect, and the indirect effect with 5.000 resamples and bias-corrected 95% confidence intervals. This method does not assume normality of sampling distribution and has been proven to have the highest power and the best type I error control [43]. Statistical significance of the mediation effect was determined if the confidence interval of the indirect effect excluded zero, indicating that the indirect effect was significant and was different from zero [42]. All results were considered significant at the level *p* < 0.05.

## 3. Results

### 3.1. Descriptive Analysis

From 200 randomly selected individuals with the post-COVID-19 condition screened for participation, after verifying that they fulfilled all criteria, 146 (53.4% women, age: 57.5 ± 12 years) patients were finally included and analyzed. The reason for exclusion of the 54 participants was because they did not report post-COVID pain. Participants were assessed at a mean of 18.8 ± 1.8 months after hospitalization. Table 1 shows the descriptive data of the sample.

### 3.2. Mediation Analysis

The regression coefficients of the mediation models can be observed in Table 2. The first model of mediation revealed that higher CSI score was significantly associated with higher PSQI scores (β = 0.39, *p* < 0.01), and higher PSQI scores were also significantly associated with lower scores in HRQoL (β = −0.26, *p* < 0.05).

The total effect of sensitization-associated symptoms (CSI) on quality of life was significant (β = −0.19, *p* < 0.05), showing a negative relationship. However, the direct effect of sensitization-associated symptoms on quality of life was not significant after including sleep quality in the model. In fact, the indirect effect of sensitization-associated symptoms on quality of life through sleep quality was statistically significant (β = −0.10, 95% CI −0.1736, −0.0373, Figure 1).

The second model of mediation showed that higher HADS-D scores were significantly associated with higher PSQI scores (β = 0.35, *p* < 0.01), and that higher PSQI scores were significantly associated with lower scores in HRQoL (β= −0.27, *p* < 0.01). The total effect of depression on quality of life was significant (β = −0.17, *p* < 0.05), indicating that higher depressive levels were associated with worse quality of life. Again, the direct effect of depression on quality of life was not significant after introducing sleep quality in the model, since the indirect effect of depression on quality of life through sleep quality was statistically significant (β = −0.09, 95% CI −0.1789, −0.0314, Figure 1).

Finally, data obtained in the third mediation model pointed out that higher HADS-A scores significantly predicted higher PSQI scores (β = 0.31, *p* < 0.01), and higher scores in PSQI were again significantly associated with lower scores in HRQoL (β = −0.28, *p* < 0.01). In turn, neither the total nor the direct effect of anxiety on quality of life was statistically significant. Finally, an indirect effect of anxiety on quality of life through sleep quality was statistically significant (β = −0.09, 95% CI −0.1648, −0.0337, Figure 1).

## 4. Discussion

The current study demonstrated that sleep quality mediates the relationship between symptoms associated with sensitization and mood disorders, i.e., anxiety/depressive symptoms, with HRQoL in individuals who have been hospitalized due to SARS-CoV-2 acute infection and suffering from long-term post-COVID-19 pain.

Previous studies suggested the presence of sensitization-associated symptoms in patients with the post-COVID-19 condition [21,22]. These studies used the CSI for evaluating these associated symptoms; however, the exclusive use of the CSI score for identifying sensitization in people with chronic pain is not recommended because this PROM overlaps with the psychological variables, such as emotional stress [44], and because only one PROM is not able to capture the complexity of central sensitization, a process involving excitability of the central nervous system [45]. We found that sensitization-associated symptoms (e.g., CSI) and depressive symptoms (i.e., HADS-D), but not anxiety symptoms (e.g., HADS-A), had direct effects on HRQoL in people with post-COVID-19 pain. Interestingly, all the effects on HRQoL from these variables were mediated by sleep quality.

These results would agree with the assumption that the CSI score can exhibit an overlap with the emotional stress construct, particularly depression, since both sensitization-associated symptoms and depressive levels exhibited similar direct effects on HRQoL, but their effect was mediated by the quality of sleep. The association between depression, sleep quality, and sensitization-associated symptoms is based on the fact that depression and sleep disturbances are able to trigger hyperalgesic responses in the nervous system via supraspinal pain mechanism and emotional modulation of pain [31,46]. Another hypothesis could be the affectation of the renin–angiotensin system, hence affecting the central nervous system, by the virus itself [47].

The relevance of depression as a factor associated with chronic pain is supported in the literature [48]. Additionally, the relevance of depressive symptoms in people with the post-COVID-19 condition is also clear in the literature [49]. The presence of depressive symptoms has been associated to higher risk of other post-COVID-19 symptoms such as fatigue [50] or dyspnea [30]. This interaction between physical and emotional post-COVID-19 symptoms would explain a worse HRQoL experienced by the patients. Potential psychopathological mechanisms underlying depressive symptoms in subjects with the post-COVID-19 condition are mainly related to the inflammation triggered by an exaggerated immune response to the viral acute infection (i.e., cytokine storm) and to the persistent psychological burden during (e.g., severity of the disease, or hospitalization) and after (e.g., isolation, post-traumatic stress, or uncertainty about prognosis) the acute infection [49]. Since anxiety and depressive symptoms can be present up to several years after the infection [51], continuous monitoring of this symptom will be important for determining the evolution of HRQoL in these patients. Nevertheless, the effects of depressive levels on HRQoL were mainly mediated by the quality of sleep.

The most important finding of this study is the role of sleep quality in individuals with post-COVID-19 pain. That sleep problems are prevalent in people with the post-COVID-19 condition is supported by available data [52]. Patients with the post-COVID-19 condition report greater difficulty falling asleep at their desired bedtime and also waking up at their desired wake time [53]. El Sayed et al. observed that sleep disturbances in post-COVID-19 patients were associated with physical and mental aspects of quality of life [54]. However, this study only analyzed the linear association between sleep quality and HRQoL. Our results further support a role of sleep in patients with post-COVID-19 pain. Although poor sleep quality has been associated with increased depressive and anxiety levels in post-COVID-19 patients [55], our study has shown that sleep quality indirectly mediated the relationship between these emotional disorders and HRQoL in those with pain symptomatology. The direction of this relationship can be bidirectional since depressive or anxiety symptoms may induce worse sleep quality, but poor sleep quality can perpetuate or potentiate these emotional disorders. The mediation effect of worse sleep quality in HRQoL may be related to associated repercussions, such as tiredness or lack of energy [56]. Independently of the direction of this association, current data suggest that poor sleep quality, depressive levels, anxiety levels, and sensitization-associated symptoms display complex relationships, and all likely influence each other in a vicious cycle in people with the post-COVID-19 condition.

The results from the current study have potential clinical implications for treatment of patients with post-COVID-19 pain. First, management of sensitization-associated symptomatology and depression may induce an improvement in HRQoL (due to their direct effects). In fact, evidence supports the use of resistance (e.g., 1–2 sets of 8–10 repetitions at 30–80% of 1RM) and aerobic (e.g., 5 to 30 min at moderate intensity) exercise programs for the management of HRQoL and emotional stress in patients with the post-COVID-19 condition [57]. A rationale for this improvement would be that exercise effects are mainly mediated by the central nervous system, and, accordingly, an effect in the nervous system of a patient with the post-COVID-19 condition would be expected [58]. Second, direct treatment of sleep could improve HRQoL in these patients (due to their indirect effect of HRQoL). Nevertheless, a meta-analysis identified that cognitive behavioral therapy was effective for managing sleep in people with chronic pain, but its direct effect on HRQoL and psychological health was not clear [59]. Accordingly, treatment of HRQoL in individuals with post-COVID-19 pain symptoms should be multimodal by targeting all variables identified in the current study, i.e., sensitization-associated symptoms, depressive levels, anxiety levels, and sleep quality. Obviously, those therapeutic strategies targeting one of these factors will have an indirect effect in those interconnected variables. Future studies evaluating the treatment strategies for patients with post-COVID-19 pain should include the topics identified in the current study.

The current study has some limitations. First, current data can only be applicable to previously hospitalized COVID-19 survivors. We do not currently know if the same associations will be seen in non-hospitalized COVID-19 survivors. However, we did not collect data regarding COVID-19 severity and other factors associated with hospitalization. Second, we used a cross-sectional design, and the mediation analyses were correlational, precluding any causal inference. Third, we did not assess the presence of depressive and anxiety symptoms at the acute phase of the infection or before the infection; accordingly, we cannot exclude the potential presence of these emotional disorders before the acute infection. Further, the levels of depression and anxiety observed in our sample of long haulers could be considered small. Fourth, the use of the CSI for assessing the presence of sensitization-associated symptoms may be confounded by coping/emotional traits. Finally, we did not analyze the presence of post-traumatic stress symptoms, which is common in people exposed to the trauma resulting from an infectious disease outbreak such as COVID-19 [60]. We do not currently know the role of post-traumatic stress in HRQoL in individuals with long-COVID. Future studies assessing the role of post-traumatic stress in post-COVID-19 are needed.

## 5. Conclusions

The current study identified that sleep quality mediates the relationship between sensitization-associated symptoms, anxiety, and depressive levels with health-related quality of life in previously hospitalized COVID-19 survivors with post-COVID-19 pain. Management of cognitive and emotional symptoms in individuals with post-COVID-19 pain could exhibit a direct and indirect effect on health-related quality of life.

## Figures and Tables

**Table 1:** Means and standard deviations of study variables.

	Mean	SD
Central sensitization (CSI, 0–100)	33.9	17.25
Depression (HADS-D. 0–21)	5.0	4.3
Sleep quality (PSQI, 0–21)	8.1	4.3
Anxiety (HADS-A, 0–21)	5.3	4.2
Quality of life (EQ-5D-5L, 0–1)	0.8	0.2

CSI: Central Sensitization Inventory; EQ-5D-5L: EuroQol-5D questionnaire; HADS: Hospital Anxiety and Depression Scale; PSQI: Pittsburg Sleeping Quality Index; SD: standard deviation.

**Table 2:** Regression coefficients of mediation analyses.

			Coefficients					95% Confidence Interval	
	Independent Variable	Dependent Variable	β	SE	t	p	R^2^	Lower Limit	Upper Limit
**Model 1**									
	CSI	PSQI	0.39	0.01	5.08	0.001 **	0.39	0.0592	0.1344
Total effect	CSI	EQ-5D-5L	−0.19	0.00	−2.44	0.01 *	0.19	−0.0042	−0.0004
Direct effect	CSI	EQ-5D-5L	−0.09	0.00	−1.11	0.26	0.31	−0.0031	0.0009
	PSQI		−0.26	0.00	−3.05	0.001 **		−0.0205	−0.0044
Indirect effect			−0.10	0.03				−0.1736	−0.0373
**Model 2**									
	HADS-D	PSQI	0.35	0.07	4.53	0.001 **	0.35	0.1988	0.5058
Total effect	HADS-D	EQ-5D-5L	−0.17	0.00	−2.12	0.03 *	0.17	−0.0158	−0.0006
Direct effect	HADS-D	EQ-5D-5L	−0.07	0.00	−0.91	0.36	0.30	−0.0116	0.0043
	PSQI		−0.27	0.00	−3.21	0.001 **		−0.0209	−0.0050
Indirect effect			−0.09	0.03				−0.1789	−0.0314
**Model 3**									
	HADS-A	PSQI	0.32	0.08	4.00	0.001 **	0.31	0.1625	0.4795
Total effect	HADS-A	EQ-5D-5L	−0.14	0.00	−1.73	0.08	0.14	−0.0147	0.0010
Direct effect	HADS-A	EQ-5D-5L	−0.05	0.00	−0.62	0.53	0.30	−0.0105	0.0054
	PSQI		−0.28	0.00	−3.38	0.001 **		−0.0213	−0.0056
Indirect effect			−0.09	0.03				−0.1648	−0.0337

SE: standard error; * *p* < 0.05; ** *p* < 0.01; CSI: Central Sensitization Inventory; EQ-5D-5L: EuroQol-5D questionnaire; HADS: Hospital Anxiety and Depression Scale; PSQI: Pittsburg Sleeping Quality Index.

## Data Availability

Not applicable.
